# Spatial modelling of malaria prevalence associated with geographical factors in Houet province of Burkina Faso, West Africa

**DOI:** 10.1007/s10708-022-10692-7

**Published:** 2022-08-01

**Authors:** Abdoul Azize Millogo, Lassane Yaméogo, Daouda Kassié, François de Charles Ouédraogo, Charles Guissou, Abdoulaye Diabaté

**Affiliations:** 1 Institut des Sciences des Sociétés, Ouagadougou, Burkina Faso; 2 Université Joseph Ki-Zerbo, Ouagadougou, Burkina Faso; 3 Centre de Coopération Internationale en Recherche Agronomique pour le Développement (CIRAD), UMR ASTRE (Animal, Santé, Territoires, Risques, Ecosystèmes), Montpellier, France; 4 Institut de Recherche en Sciences de La Santé/Centre Muraz, Bobo-Dioulasso, Burkina Faso

**Keywords:** Malaria, Burkina Faso, Ordinary least squares (OLS), Vegetation, Rainfall, Temperature, Distance to water, Soil permeability

## Abstract

Malaria is a permanent threat to health in western Burkina Faso. Research has shown that geographical variables contribute to the spatial distribution in its transmission. The objective of this study is to assess the relationship between malaria prevalence and potential explanatory geographical variables in the Houet province in Burkina Faso. Statistics on malaria prevalence registered by health centres in the Houet province in 2017 and potential geographical variables identified through a literature review were collected. An Ordinary Least Squares (OLS) regression was used to identify key geographical variables and to measure their association with malaria while the Getis Ord Gi* index was used to locate malaria hotspots. The results showed that average annual temperature, vegetation density, percentage of clay in the soil, total annual rainfall and distance to the nearest waterbody are the main variables associated with malaria prevalence. These variables account for two-thirds of the spatial variability of malaria prevalence observed in Houet province. The intensity and direction of the relationship between malaria prevalence and geographical factors vary according to the variable. Hence, only vegetation density is positively correlated with malaria prevalence. Average temperature, for soil clay content, annual rainfall and for distance to the nearest water body are negatively correlated with the disease prevalence. These results show that even in an endemic area, malaria prevalence has significant spatial variation. The results could contribute to the choice of intervention sites, as this choice is crucial for reducing the malaria burden.

**Supplementary Information:**

The online version contains supplementary material available at 10.1007/s10708-022-10692-7.

## Background

Malaria is threatening 40% of the world's population (WHO, 2015). The number of malaria cases was estimated to 241 million in 2020. Sub-Saharan Africa is a large endemic area, with most of the worldwide cases (95%) occurring in Africa (WHO, 2021) due to favourable natural conditions and poverty (WHO, 2015). Taking into account the new methodology for calculating the deaths toll in children under five being caused by malaria (Perin et al., 2022), the number of deaths due to malaria has been estimated at 627 000 in 2020. The WHO African Region continues to bear the largest burden with 96% of all malaria deaths in 2020 (WHO, 2021).

Burkina Faso is one of the six countries with the highest number of malaria cases and deaths in the world (WHO, 2021). Statistics from 2020 show that Burkina Faso has registered 12,410,000 cases of malaria and 21 100 deaths from malaria (WHO, 2021). The country is subdivided into three (3) malaria transmission zones: a short seasonal transmission zone in the north, a long seasonal transmission zone in the centre and a permanent transmission zone in the south.

The province of Houet is located within the permanent transmission zone (IGN France International & IGB, 2015; Mouchet et al., 1993; Zon & Barrère, 2002). The average prevalence of malaria was 47.39% or 715,920 cases in the Houet province for a population of 1,510,638 in 2020 (Ministère de la Santé, 2020; Ministère de l’Economie des Finances et du Plan, 2022). These malaria cases are distributed as follows: 692,826 cases of simple malaria and 23,094 cases of severe malaria. On the same period, the country registered 250 death due to severe malaria (Ministère de la Santé, 2020).

Like in most malaria-endemic countries, malaria control is carried out in Burkina Faso through vector control and chemoprophylaxis. Thus, since 2010, national campaigns have been carried out by the Health ministry to distribute massively mosquito net to the population. To date, the insecticide-treated bed net (ITN) use rate is about 76% on the country scale compared to 80% in the Houet province (INSD, 2018). ITN distribution campaigns are supported by indoor residual spraying (Ministère de la santé, 2016). Chemoprophylaxis is conducted through seasonal malaria chemoprevention (SCP) for children from three (3) to fifty-nine (59) months of age and Intermittent preventive malaria treatment using sulfadoxine-pyrimethamine (IPTp-SP) for pregnant women (Ministère de la santé, 2016). More than 80% of the SCP targets were given treatment (Ministère de la santé, 2016) and at least 57% of women in the country as well as in the Houet province receive three doses of IPTp-SP (INSD, 2018). In addition, 82,5% of malaria cases were treated with ACT, 3,3% with artesunate or arthemeter injectable. At the provincial scale, these statistics reach 94,0% and 4,9% (Ministère de la Santé, 2020).

Despite these control measures, malaria remains the main cause of medical consultation, hospitalization, and death in Burkina Faso. In-depth studies are required to improve the understanding of the persistence of the disease. The results of such studies can guide field intervention programs and improve their efficiency.

Several studies have shown the role of geographical factors (climate, vegetation, soil, surface water, etc.) in malaria development and transmission. Rainfall and temperature are the two main parameters most associated with the disease (Kakmeni et al., 2018; MARA/ARMA, 1999) because they directly influence the vector's reproductive cycle (Carnevale & Robert, 2009) and set the pace of the mosquito and plasmodium development cycle (Adigun et al., 2015; Nkurunziza et al., 2010; Samadoulougou et al., 2014). However, the role of other factors is not negligible. Vegetation can be used as a resting and protective site against adverse weather conditions, or as a barrier to adult mosquito movement (Adigun et al., 2015; Minale & Alemu, 2018; Rageau & Adam, 1953; Samadoulougou et al., 2014). Topography and nature of the soil, through the retention time of water, can influence larval formation pools in puddles that serve as breeding sites for mosquitoes (Carnevale, & Robert, 2009; Dembélé & Somé, 1991; Hasyim et al., 2018; Kazembe et al., 2006). These breeding sites add to the local malaria transmission risk because mosquitoes that emerge from them develop and breed nearby (Epopa et al., 2019). The probability of malaria transmission is a function of distance from larval habitats (Kleinschmidt et al., 2001; Samadoulougou et al., 2014; Tuyishimire et al., 2016).

Geographical factors appear to be amongst the main factors that modulate the likelihood of malaria transmission. Their relationship with the pathology can be multidirectional through impacts on the vector and the parasite, hence the need to understand, for any ecosystem, how these relationships works to adapt control policies. Models which include these variables while measuring the intensity of their relationship with the disease are necessary for disease surveillance (WHO, 2015).

The province of Houet is located in the western Burkina Faso, most watered part of the country with a relatively dense hydrographic network and vegetation cover. Despite these quasi homogeneous conditions, the observed prevalence is quite heterogeneous. Recent research has shown that malaria prevalence can be heterogeneous even on a fine scale (Ndiaye et al., 2020). The objective of this study is to model the relationship between geographical variables and malaria prevalence in the Houet province. The aim is to determine the direction and strength of the relationship between disease and geographical variables within the Houet province.

## Methods

### Study area

**Fig. 1:**
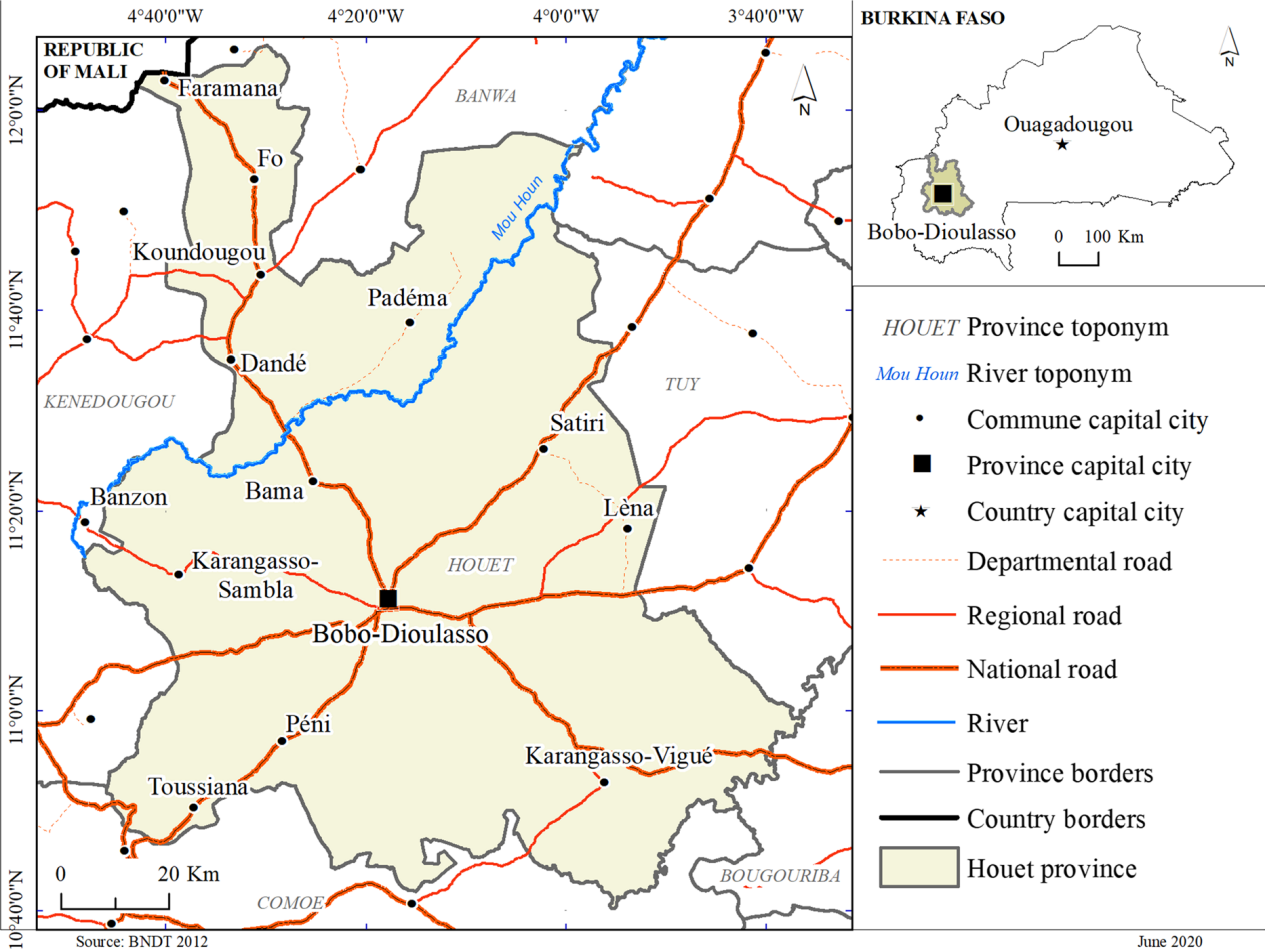
Location of the province of Houet. Source: Base Nationale des Données Topographique (BNDT) 2012

The study area is the Houet province, located in the Hauts-Bassins region of western Burkina Faso. It includes the urban municipality of Bobo-Dioulasso, the country's second largest city, 12 rural communes, 207 villages, and covers an area of 11,582 km^2^ (IGB, 2015) (Fig. 1). The choice of the province as the study area is justified by its location in the continuous transmission zone. This allows a better assesment of the intensity of the relationship between the disease and the geographical variables. The results obtained could also be extrapolated to areas with similar physical characteristics.

The Houet province is characterized by a tropical climate marked by two main seasons: a wet season which lasts from May to October and a dry season which extends from November to April. The average annual temperatures are between 25 °C and 30 °C. The region benefits from an average annual rainfall ranging between 800 and 1100 mm but this is not evenly distributed geographically (Malo, 2017). In this environment, the vegetation is mainly composed of savannah, which covers 31.19% of the total area of the province. This vegetation is supported by six classes of soil: organic soils with sesquioxide and rapid mineralization, Mull soils, hydromorphic soils, fersiallitic soil, raw mineral soils and vertisols (Malo, 2017).

Houet province is covered by the Health Districts of Dafra, Dô, Lena, Dandé and Karangasso Vigué (Ministère de la santé, 2018). These districts have 104 Health and Social Promotion Centres, two standard medical centres, two medical centres with surgical units and one university hospital.

### Study population

It is estimated that the province had a population of 1,510,638 in 2020. It is distributed in the six health districts of the province as follows 36.3% in the Do health district, 23.1% in the Dafra health district, 18.4% in the Dandé health district, 10.0% in the N'Dorola district, 7.1% in the Karangasso Vigué district and 5.1% in the Léna health district (Ministère de l’Economie des Finances et du Plan, 2022). The populations are still disaggregated in the health care areas (HCA) that are the observation units in this study. Lack of administrative boundaries separating the HCA does not allow accurate population estimate.

The literature on modelling malaria using geographical and environmental variables allows the selection of potential variables associated with malaria transmission: minimum temperature, maximum temperature, average temperature, total annual rainfall, vegetation density, clay concentration in the soil, elevation, distance from the nearest water body, distance from the nearest health facility, population density (Hasyim et al., 2018; Kleinschmidt et al., 2000; Kleinschmidt et al., 2001; Nkurunziza et al., 2010; Rouamba et al., 2019; Samadoulougou et al., 2014; Tuyishimire et al., 2016). In this present study, waterbody is composed of the lakes, ponds and rivers listed in the BNDT and NBDOT 2012. The characteristics of the selected variables are presented in Table 1.

**Table 1:** Characteristics and sources of data for potential explanatory variables

Data type	Variable extracted	Date	Resolution/ Spatial scale	Provider/Source
Malaria data: Location of health facilities; malaria prevalence rates	Malaria prevalence (%)	2017	Health care area	Direction régionale de la santé des Hauts-Bassins (Regional Health bureau)
Coordinates of health care facilities	Distance to the nearest health care facility (m)	2011 (update 2017)	Province	Direction régionale de la santé des Hauts-Bassins (Regional Health bureau)
Demographic data	The population of the health care area andPopulation density (people per square km)	2006 (Projection to 2017)	Village	Institut national de la statistique et de la démographie (INSD) 2006 (National Institute of Statistics and Demography)
Land use: Vegetation, floodplains, waterbodies	Distance to the nearest Waterbody (m); Vegetation density (score)	2010	Province	Nouvelle Base de Données d’Occupation des Terres (NBDOT) de l'Institut géographique du Burkina (IGB) (New Land Use Database) https://www.ignfi.fr/fr/portfolio-item/occupation-des-terres-burkina-fao/
Clay concentration in the soil	Soil permeability (%)	2015	250 m	International Soil Reference and Information Centre (ISRIC) https://www.isric.org/
Temperature data	Mean temperature variability map (°C)	1970–2000	1 km	worldClim version 2.0 http://worldclim.org/version2
Rainfall data	Total rainfall variability map (mm)	1987*–*2016	Province	Agence nationale de la météorologie (ANAM). (National Meteorological Agency)
Elevation	Elevation (m)	*2006–2011*	Province	*Advanced Land Observing Satellite (ALOS) from* Open topography https://opentopography.org/

Source: A. A. MILLOGO. 2018

### Data collection

The data on malaria were collected from the Regional Health Direction of the Hauts-Bassins. They were collected during the year 2017, from January to December from the health facilities: CSPS, CM, MCA and CHU. These data assess number of cases for both simple and severe malaria, the number of deaths, information on awareness sessions as well as the number of people reached. Malaria cases were confirmed by rapid diagnostic test (RDT) or thick blood smear and registered in the consultation registry (Ministère de la Santé, 2020).

### Data pre-processing

Due to the variety of sources, formats and resolutions, the data have been pre-processed. This treatment allowed us to harmonize the data format in order to prepare the modelling. Thus, Thiessen polygons were plotted to delineate the HCA of each health care facility.

Then, the contours of the Thiessen polygons define the area closest to each health centre considering all other surrounding health centres. This technique assumes that patients use the nearest health centre despite the results of studies which have shown that the use of care follows other spatial logics (Cisse, 2007). This choice is justified by the non-availability of a layer of HCA boundaries. Then, the total number of malaria cases as well as the total population of villages within the HCA were reported to the health centre layer to calculate malaria prevalence. Finally, potential explanatory variables were prepared. Thus, the land-use units contained in the New Land Use Database (NBDOT) were reclassified according to the vegetation density using a visual interpretation of the photographs contained in the database user guide. The classification was done according to a score assigned to each vegetation class and the results were rasterized (Table 2).

**Table 2:** Level of vegetation density in land use classes from the NBDOT

NBDOT land use unit	Vegetation density	Score
Bare rock	Low	1
Bare soil (eroded, bare, cuirass, etc.) dune and sand		
Water surface		
Residential area		
Wet area	Moderately Low	2
Irrigated crop		
Rainfed culture and agroforestry territory		
Tree savannah	Moderately high	3
Shrubby and grassy savannah		
Clear forest	High	4
Gallery forest		
Plantation forest		
Orchard		

Using the Near (Analysis) tool in ArcGIS 10.4®, a distance matrix was generated between each village in the study area and the nearest waterbodies. This matrix was joined to the health facility layer and the water point to generate a map of distances (in meters) to health centres. The same operation was performed on the localities and health facilities layer to generate a map of distances to the nearest health facility. For temperature, elevation and soil clay content, the global scale rasters downloaded were reprojected to WGS84_UTM_Zone_30N. Then, the Houet province zone was extracted to produce the corresponding maps. Due to its deterministic nature, total annual precipitation for each station was calculated and interpolated by the kriging method (Gunarathna et al., 2016) to produce the map.

**Fig. 2:**
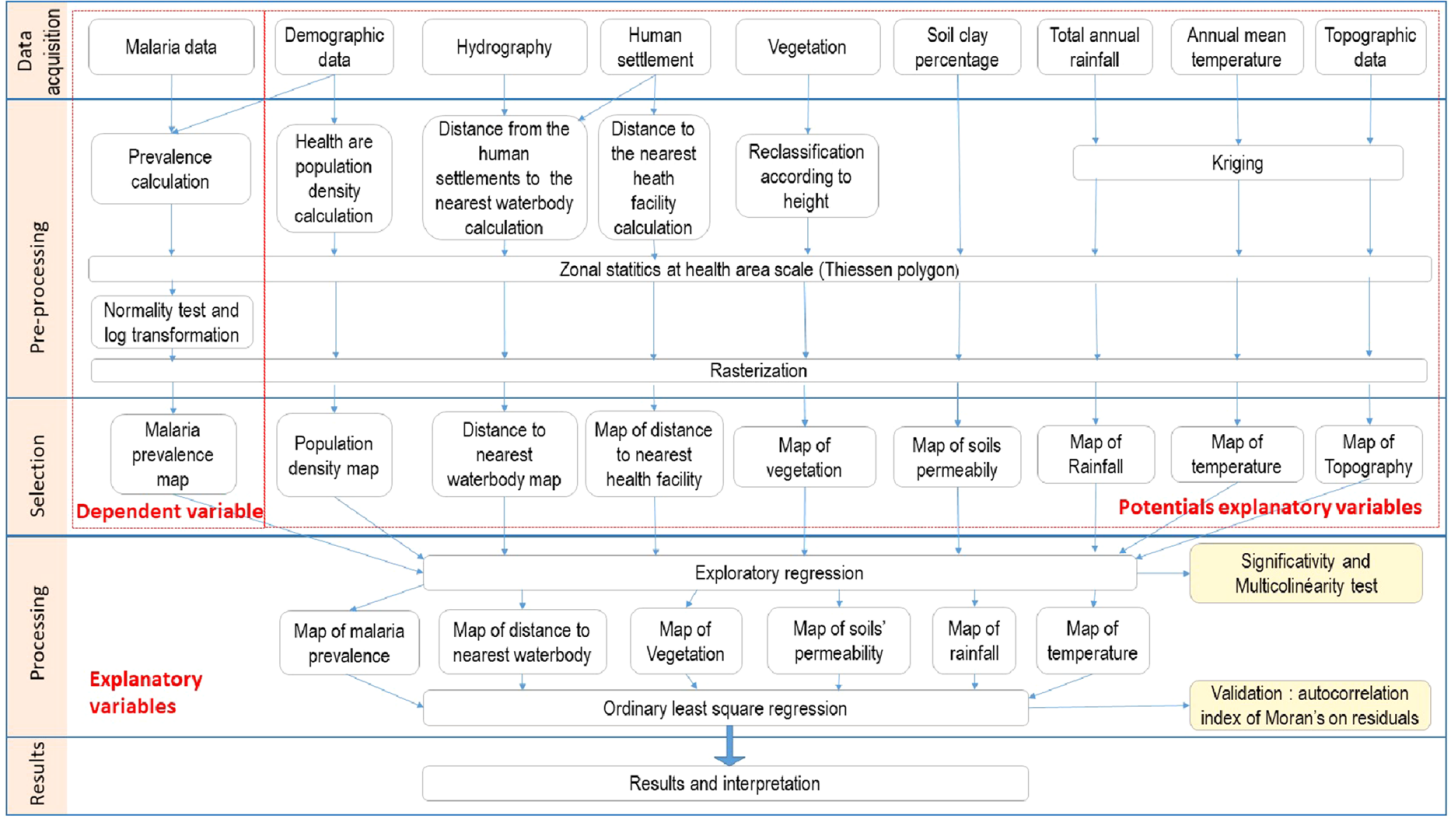
Modelling process. This figure shows the data preprocessing process, the selection of key variables and the modelling process. Source: A. A. MILLOGO, 2018

The rasters of the generated explanatory variables were projected to the WGS84_UTM_Zone_30N coordinate system and resampled at a spatial resolution of 100 m and their average values were plotted in the health facilities layer. The average values of each variable in the health facility layer were calculated using the Euclidian Allocation tool and the Extract Multiple Values (Spatial Analyst) tool was used to report the previously calculated mean values in the health facility layer. Figure 2 summarizes the data preprocessing steps.

### Data processing and modelling framework

The modelling process consisted in two steps: the first one is the selection of potential explanatory variables and second one is the determination of the direction and strength of the relationship between malaria prevalence and the identified explanatory variables. An Exploratory Regression (ER) with ArcGIS Exploratory Regression (Spatial Statistics tools) was performed on the potential explanatory variables of vegetation density, annual precipitation, average annual temperature, percentage of clay in soils, elevation, distance to the nearest water body, population density, and distance to the nearest health centre. The objectives of this step were, firstly, to identify the variables that are statistically correlated with malaria prevalence (p-value < 0.05), and secondly, to find the model with the least variables and the highest coefficient of determination (R^2^). Note that the ArcGIS® ER tool constructs Ordinary Least Squares (OLS) regression models using all possible combinations of potential explanatory variables and evaluates which models pass the necessary controls (Eq. 1). Linear least squares regression formula

1 $$y = \beta_{0} + \beta_{1} X_{1} + \beta_{2} X_{2} + \beta_{3} X_{3} + \cdot \cdot \cdot + \beta_{n} X_{n} + \varepsilon$$

*y: the dependent variable*

β: regression coefficient.

x: explanatory variable.

ε: residual.

The ER gives two types of output. The first is a table presenting the correlation coefficients and the p-values allowing the identification of the variables statically associated with malaria and the second is a table giving the general diagnosis of the constructed model. The latter gives the values of the Multiple R-Squared [d], Adjusted R-Squared [d], Joint F-Statistic [e], Joint Wald Statistic [e], Koenker (BP) Statistic [f] et Jarque–Bera Statistic [g] to judge the performance of the model under construction.

Since the ER results do not give the specific contribution of each of the selected variables, an OLS regression (using ArcGIS Ordinary Least Squares tool) was as the second step of the modelling process. OLS regression is used to measure and understand the relationships between two or more entity attributes at a specific location, and also to predict where another event is likely to occur (https://pro.arcgis.com/fr/pro-app/tool-reference/spatial-statistics/ordinary-least-squares.htm).

### Validation of the results

The performance of the selected model was evaluated through several parameters generated either by the ER analysis or by the OLS analysis. A passing model is composed of variables that are statically associated with malaria prevalence (p < 0.05) with Variance Inflation Factor (VIF) values (> 7.5), meaning that there is no collinearity between the selected variables. The model with R^2^ and the highest Akaike Information Criterion (AICc) is the one that best fits the data. The joint F[e], Wald and Koenker (BP) statistics are used to determine the overall significance of the model. When this Wald and Koenker (BP) test is statistically significant (p < 0.01), the modelled relationships are not consistent (either because of non-stationarity or heteroskedasticity). Finally, the Jarque–Bera statistic [g] is used to evaluate the normality of the residuals. When this test is statistically significant (p < 0.05), the model predictions are biased. The Moran autocorrelation test (Moran index) was performed on the residuals to check the Jarque–Bera statistic [g]. In the interpretation of the table of correlations between the dependent variable and the explanatory variables, the coefficient [a] represents the intensity and type of relationship (positive or negative) that the variable has with the dependent variable. The probability fields [b] and Robust_Pr [b] fields present the p-values of the relationship between the two types of variables. When the value of the Probability [b] field is not significant and that of Robust_Pr [b] is significant, the variable is considered to be statistically correlated with the dependent variable. (ESRI, https://desktop.arcgis.com/en/arcmap/10.5/tools/spatial-statistics-toolbox/how-ols-regression-works.htm). The steps in the process are presented in Fig. 2. Finally, an analysis of hotspots and cold spots using Getis -Ord Gi* tools on the predicted prevalence values were carried out. A hotspot is an area with a strong positive correlation between the explanatory variable and malaria prevalence, while a cold spot is an area with a strong negative correlation between the explanatory variable and malaria prevalence. The Gi* statistic makes it possible to identify hotspots when Gi* is positive and statistically significant (< 0.05) and cold spots when it is negative and statistically significant (< 0.05).

## Results

### Mapping the prevalence of malaria in Houet province

The province of Houet recorded 491,098 cases of malaria in 2017 for an estimated population of 1,386,433 people. This corresponds to an average prevalence of 35.42%.

**Fig. 3:**
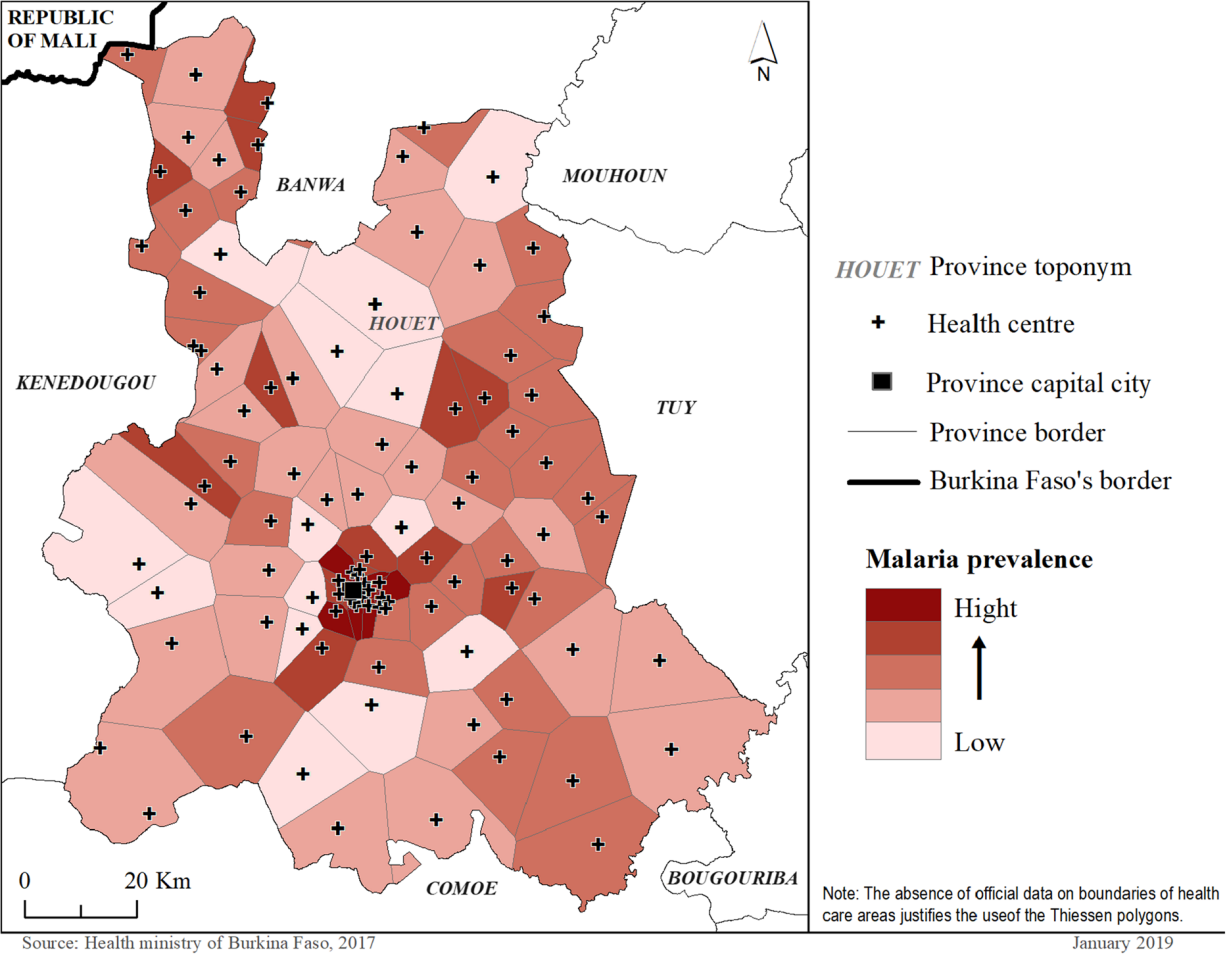
Distribution of the prevalence of malaria in each of the health areas. Source: Ministère de la santé Burkina Faso 2017; Base Nationale des données Topographique (BNDT) 2012 of Institut Géographique de Burkina (IGB)

Morans Index shows and Index of − 0.005; a Z score of 0.114 and a p-value of 0.9 showing that apart from the extreme values observed in the city of Bobo-Dioulasso, the HCA with high prevalence are randomly distributed throughout the province (Fig. 3).

### Selection of key variables

According to the exploratory least squares regression, the best performing model is composed of vegetation height, total annual precipitation, average annual temperature, percentage of clay in the soil and distance to the nearest water body. These variables are statistically associated with malaria prevalence (p < 0.05). The diagnosis of exploratory least squares regression is presented in Table 3.

**Table 3:** Diagnosis of exploratory least squares regression

Number of Observations:	97	Akaike's Information Criterion (AICc) [d]:	273.40
Multiple R-Squared [d]:	0,64	Adjusted R-Squared [d]:	0.62
Joint F-Statistic [e]:	32,82	Prob (> F), (5,91) degrees of freedom:	0.00*
Joint Wald Statistic [e]:	125,22	Prob (> chi-squared), (5) degrees of freedom:	0.00*
Koenker (BP) Statistic [f]:	5,84	Prob (> chi-squared), (5) degrees of freedom:	0.32
Jarque–Bera Statistic [g]:	2,06	Prob (> chi-squared), (2) degrees of freedom:	0.36

**Fig. 4:**
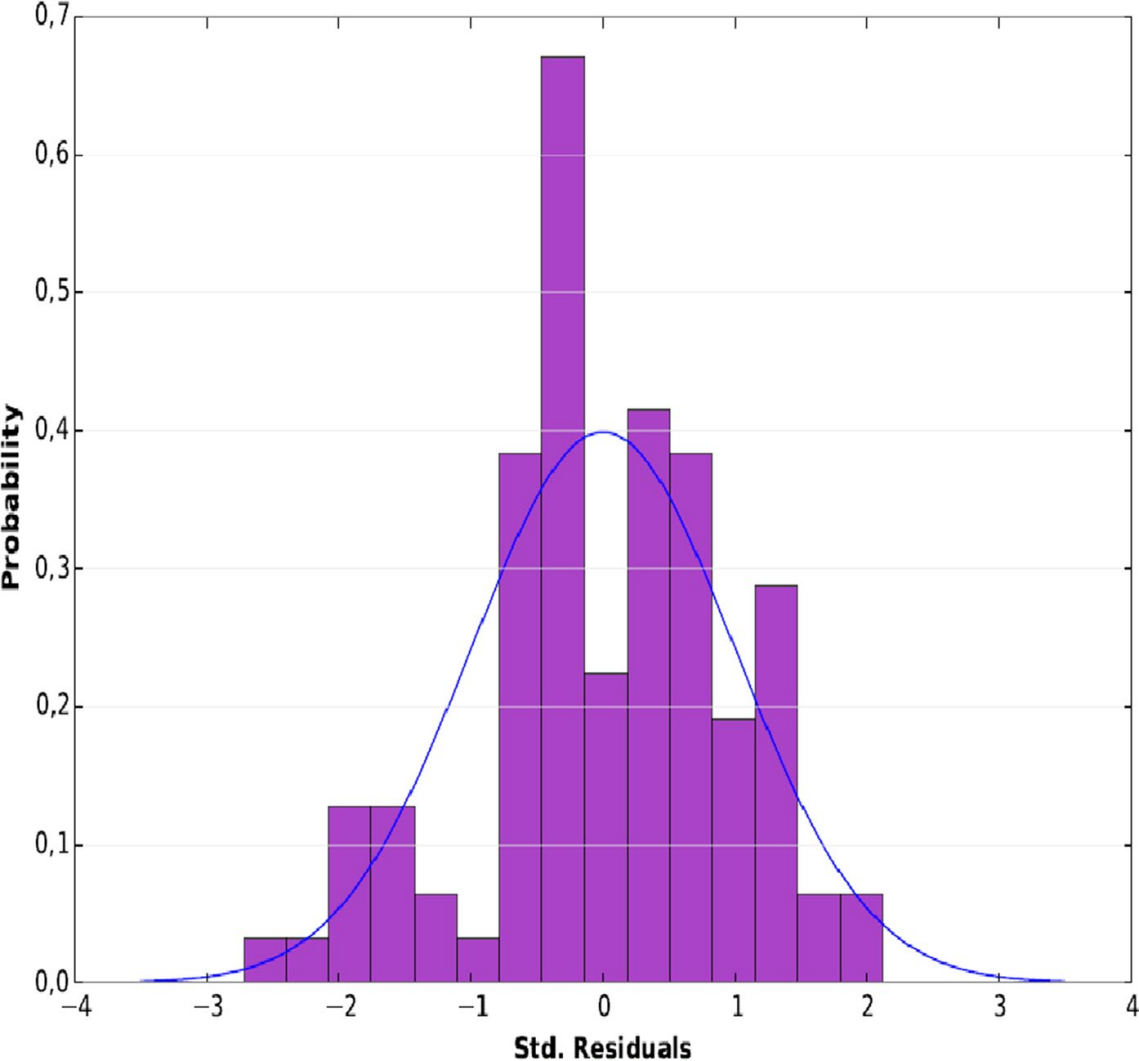
Histograms of standardised residuals. Source: A. A. MILLOGO. 2018

The Adjusted R^2^ value shows that the variables account for 63% of malaria prevalence in the study area. The joint F statistic [e] and the joint Wald statistic [e] (p-value < 0.05) show that this is a significant model. According to the probability value (0.32) of the Koenker (BP) Statistic [f], the explanatory variables of the model have a consistent relationship with the dependent variable both in geographical space (stationarity) and in data space (heteroskedasticity). The Jarque–Bera statistics is also not statistically significant (0.38), which proves that all the variables are related to the prevalence of malaria in the Houet province and that no key variable is missing from the model. This statistic also attests that the model's residuals are not spatially autocorrelated. It was confirmed by the Moran's autocorrelation test (Moran's index) carried out on standard errors (StdError). This is illustrated in Fig. 4, showing a Z score of 0.98 for a p-value of 0.33, proof that there is no spatial aggregation. If the almost correlation between the selected variables and malaria prevalence is significant, it is important to understand the intensity of the association and the location of areas of high correlation with malaria prevalence.

### Assessing the contribution of each key variable

The least squares regression results showed that the intensity and direction of the relationships vary from one variable to another. The correlation coefficients for the variables with a significant spatial correlation with malaria prevalence are presented in Table 4.

**Table 4:** Correlation of explanatory variables with malaria prevalence

Variable	Coefficient [a]	StdError	t-Statistic	Probability [b]	Robuste_SE	Robust_t	Robust_Pr [b]	VIF [c]
Intercept	67.86	17.53	3.87	0,00*	16.30	4.16	0.00*	–
Vegetation	1.90	0.17	11.06	0,00*	0.18	10.61	0.00*	1.99
Mean annual temperature	− 1.89	0.50	− 3.76	0,00*	0.48	− 3.92	0.00*	2.20
Soil clay content	− 0.25	0.09	− 2.73	0,01*	0.08	− 3.18	0.00*	1.39
Total annual rainfall	− 0.01	0.00	− 3.58	0,00*	0.00	− 3.76	0.00*	2.94
Distance to the nearest waterbody	0.00	0.00	− 1.92	0.06	0.00	− 2.29	0.02*	1.10

Source: A. A. MILLOGO. 2018

Vegetation density and average annual temperature are the variables with the highest correlation coefficients, indicating that these variables explain a large part of the variability of malaria prevalence in the province. Furthermore, only vegetation density shows a relatively strong positive spatial correlation with malaria prevalence, while all other variables have a negative relationship. Using the set of correlation coefficients, the malaria prevalence equation has been established (Eq. 2) Prevalence equation.

2 $$\begin{aligned} {\text{Malaria prevalence }}\left( \% \right) \, = & { 67}.{86 } + { 1}.{9}0*{\text{ Vegetation density }}\left( {{\text{score}}} \right) \, \\ & - {1}.{89}*{\text{ Temperature }}\left( {^\circ {\text{C}}} \right) \, - 0.0{1}*{\text{ Rainfall }}\left( {{\text{mm}}} \right) \, \\ & - 0.{25}*{\text{ Soil clay content }}\left( \% \right) \, \\ & - 0.0{1}*{\text{ Distance to the nearest waterbody }}\left( {\text{m}} \right) \, + \, \varepsilon \\ \end{aligned}$$

ε represent the residual of the model. It summarises the remaining 37% of the information that is not taken into account in the linear equation established.

The association between malaria and explanatory variables.

**Fig. 5:**
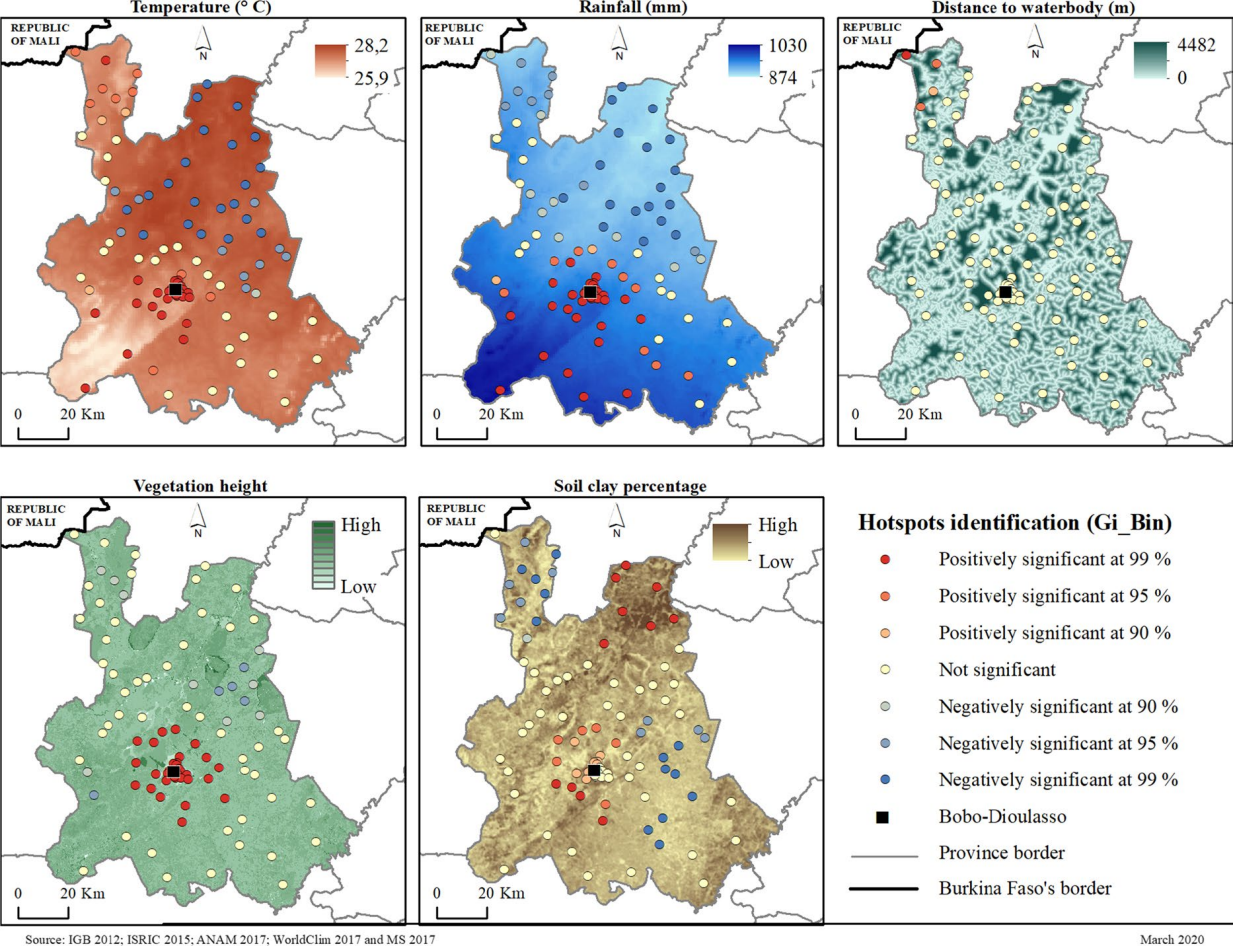
Getis -Ord Gi* carried out with the predicted prevalence of malaria according to the relationship between each variable and malaria prevalence. Source: IGB 2012, ISRIC 2015, ANAM 2017, WorldClim 2017 and Ministère de la santé 2017

Concerning areas of high correlation, it is noticeable that the intensity and their distribution also vary from one variable to another (Fig. 5).

The average temperature across the year is 27.61 °C (26.0–28.2 °C). These high temperatures are favourable for both the malaria vector and the malaria parasite (Carnevale, & Robert, 2009). The temperature hotspots are located in a sector that extends from Bobo-Dioulasso (in the centre) to the west of the province and from Bobo-Dioulasso to the south. Another hotspot zone can be identified in the north towards the Malian border. Moreover, a cold spot is located in the north and northeastern part of the province, which shows that the two variables evolve in divergent directions. This is the area where temperature has less influence on malaria transmission. The average annual total rainfall is 971 mm (874–1030 mm). The spatial distribution of rainfall shows a gradient, with the south being relatively wetter than the north. Thus, rainfall has a greater influence on the transmission of malaria in the south of the city of Bobo-Dioulasso than in the north. The average distance from localities to water bodies is 651.58 m (271.8–3579.5 m). This value indicates that the populations are close to water bodies, which are potential mosquito breeding sites. The only hotspot is identified at the Malian border. No significant relationship is observable in the rest of the provincial territory. The Malian border is in the extreme north of the province. The average vegetation density score is 6.7 (5.6–9). These values indicate a certain density of vegetation in this part of the country that is relatively well-watered. The major hotspots are concentrated around the city of Bobo-Dioulasso. Cold spots can be observed in the north-east, north and west of the province. Around Bobo-Dioulasso, the relationship is due to the low density of vegetation cover. The average clay content of the soil is 60.51% (57.8–63.7%). A high clay content confers low permeability, favoring mosquito breeding in stagnant water. Hotspots at this level can be observed where the clay contents are the highest, i.e. to the west of Bobo-Dioulasso and to the north. In contrast, cold spots are observed to the east and north, towards the Malian border. They can be overlaid on the parts of the province with low clay content, which are therefore more permeable.

## Discussion

This study identified the geographical variables correlated with malaria transmission in the Houet province and determined their relationships with the disease prevalence by using ordinary least squares (OLS) regression. It reveals that vegetation density, average annual temperature, average annual rainfall, soil clay content and distance to nearest standing water are key variables explaining the transmission of malaria in the Houet province. This method was used in Mali in the Malaria Atlas for Africa (MARA/ARMA) project with an R^2^ of 0.73 (Kleinschmidt et al., 2000), in Kenya with an R^2^ of 0.81 (Noor et al., 2009) and in Burkina Faso to model malaria risk in children under five years old with an R^2^ of 0.77 (Samadoulougou et al., 2014). An author from Indonesia used Geographically Weighted Regression (GWR) to achieve an R^2^ of 0.69 (Hasyim et al., 2018).

The western part of Burkina Faso has higher rainfall than the rest of the country. Its environmental parameters offer optimal conditions for the development and persistence of malaria. In this area, the denser the vegetation, the lower the prevalence of malaria. Dense and high vegetation can prevent the spread of *Anopheles* by providing them with a kind of screen on which they will land. This explains why an increase in mosquito density is commonly observed in newly cleared areas (Amat-Roze, 2002; Carnevale & Robert, 2009). However, studies using the Normalized Difference Vegetation Index (NDVI) in Burkina Faso, Kenya and Bangladesh concluded that malaria prevalence was associated with high Normalized Difference Vegetation Indices (NDVI) (Noor et al., 2009; Reid et al., 2010; Samadoulougou et al., 2014). In this study, temperature is negatively associated with malaria prevalence. The average temperature in the Houet province is 27.61 °C. Experimental studies have shown that the prevalence of infected mosquitoes was 16%, 8% and 6% at 27 °C, 30 °C and 32 °C respectively (Okech et al., 2004). Mosquito infection rates decrease with increasing temperature (Carnevale & Robert, 2009) and field data have confirmed that the disease is negatively associated with temperature in West Africa (Arab et al., 2014) and East Africa (Nkurunziza et al., 2010). Rainfall is favourable to malaria vectors as it feeds ponds and puddles used by mosquitoes to breed. However, beyond certain quantities of rainfall, the larvae are washed away by running water, which reduces the chances of disease transmission. The peak of transmission is observed in October when the rainfall decreases, while the rainiest month is July (Okech et al., 2007). Thus, in Burkina Faso and Burundi, malaria is positively associated with rainfall (Nkurunziza et al., 2010; Rouamba et al., 2019). In the Houet province, malaria prevalence and distance from water bodies are negatively correlated. Water bodies are breeding sites and studies have shown that mosquitoes do not move away from the breeding site when they can feed (Epopa et al., 2017). Similar observations have been made in Indonesia (Hasyim et al., 2018), where the distance from rivers and lakes was correlated with malaria prevalence, and in Rwanda, where malaria infection increases with proximity to irrigated farmland (Tuyishimire et al., 2016).

The specificity of this study lies in three key points. Firstly, this study highlighted the contribution of soil to malaria prevalence. This variable influences vector ecology by extending or shortening the lifespan of breeding sites through infiltration rate. This variable has been identified as a major risk factor in the Fayoum governorate in Egypt (Hassan et al., 2003) and as an environmental determinant of malaria in Benin (Pierrat, 2010). The nature of the substrate of the breeding sites (clay or sandy substrates or lake water without substrate) influences the vectorial competency of *An. gambiae* in relation to *P. falciparum* (Okech et al., 2007). These results are confirmed by preliminary studies (Amat-Roze, 2002; Rageau & Adam, 1953). Secondly, the study has identified malaria hotspots in the Houet province. These results are consistent with recent studies that have indicated the importance of geographical factors in identifying malaria transmission hotspots in Gambia (Ndiaye et al., 2020). It is particularly important as studies are underway in the same province to find an effective method to control residual malaria transmission (Niang et al., 2021). Thirdly, this study used prevalence data from the general population while most other studies are based on children under five years of age (Samadoulougou et al., 2014) or 10 years of age (Kazembe et al., 2006). It also takes adults into account, a group considered relatively less vulnerable to malaria. Despite their small proportion in the statistics of consultations and deaths (> 5%) (Ministère de la santé, 2018), the impact of their absence due to illness impacts on their household and the wider community in terms of decreased working time and financial loss.

Despite its specificities, this study had some limitations. Firstly, unlike previous studies in Burkina Faso and Tanzania (Kienberger & Hagenlocher, 2014; Msugupakulya et al., 2020; Rouamba et al., 2019; Samadoulougou et al., 2014), it did not integrate data on the socio-economic characteristics of populations despite malaria prevalence being a complex phenomenon that is determined by both socio-economic and physical environmental variables. This is due to the difficulty of collecting these data in all of the villages within the study area. Moreover, the relevance of using this type of data on a large scale has been questioned because of its variability within a single locality (Pierrat, 2010). This question is still relevant in the current context given the variability of these types of data. Secondly, concerning the data that were collected, the temperature data used in this study is based on a period covering the years 1970–2000, as the province is covered by a single synoptic weather station that would not allow any spatial variation to be observed. Spatial modelling based on 30 years of data (Fick & Hijmans, 2017) seems to be a reliable source to overcome this difficulty. Similarly, we used projected population data up to 2017 because the available data come from the 2006 population and housing census. Due to political instability (insurrection in October 2014 and coup d'état in September 2015), the country was unable to update the demographic data in 2016. Thirdly, the average values of malaria prevalence and independent variables were reported to the CSPS, which is the centroid of the polygons of the health area. This procedure leads to a significant loss of information and does not allow an adequate appreciation of the local variability of the disease, health seeking migration, and local associations with the independent variables. However, these limitations are perspectives for further research.

## Conclusion

This study confirmed the relevance and intensity of the relationship between several key geographical variables and malaria prevalence. The least squares regression showed a strong positive association between vegetation density and malaria prevalence, and a strong negative association between both temperature and soil clay content with malaria prevalence. Rainfall, population density and distance from water bodies play a secondary role. The correlated variables explain two-thirds of the variability of malaria in the province. Further analysis should investigate the contribution of socio-economic variables.

## Supplementary Information

Below is the link to the electronic supplementary material.

Supplementary file1 (XLSX 52 kb)

## Data Availability

All data generated or analysed during this study are included in this published article and its supplementary information files.

## Abbreviations

ANAM: Agence Nationale de la Météorologie (National Meteorological Agency)
BNDT: Base Nationale des Données Topographique (National Topographic Data Base)
ER: Exploratory Regression
GWR: Geographically Weighted Regression
IGB: Institut Géographique du Burkina (Geographical Institute of Burkina Faso)
IGN: Institut Géographique National (National Geographical Institute)
INSD: Institut National de la Statistique de la Démographie (National Institute of Statistics and Demography)
IPTp-SP: Intermittent preventive treatment of malaria in pregnancy using sulfadoxine-pyrimethamine
ISRIC: International Soil Reference and Information Centre
ITN: Insecticide-Treated bed Net
NBDOT: Nouvelle Base de Données d’Ocuppation des Terres (New Land Use Database)
NDV I: Normalized Difference Vegetation Index
OLS: Ordinary least squares
WHO: World Health Organisation
SCP: Seasonal malaria chemoprevention
